# Validation of miR-1228-3p as Housekeeping for MicroRNA Analysis in Liquid Biopsies from Colorectal Cancer Patients

**DOI:** 10.3390/biom10010016

**Published:** 2019-12-20

**Authors:** Saray Duran-Sanchon, Elena Vila-Navarro, Maria Marcuello, Juan José Lozano, Jenifer Muñoz, Joaquín Cubiella, Maria Soledad Diez, Luis Bujanda, Angel Lanas, Rodrigo Jover, Vicent Hernández, Enrique Quintero, Marta Herreros-Villanueva, Ana Carmen Martín, Rosa Pérez-Palacios, Rocio Arroyo, Antoni Castells, Meritxell Gironella

**Affiliations:** 1 Gastrointestinal & Pancreatic Oncology Group, Centro de Investigación Biomédica en Red de Enfermedades Hepáticas y Digestivas (CIBEREHD) /Hospital Clínic of Barcelona/Institut d’Investigacions Biomèdiques August Pi i Sunyer (IDIBAPS), University of Barcelona, 08036 Barcelona, Catalonia, Spain; 2 Bioinformatics Platform, CIBEREHD, 08036 Barcelona, Catalonia, Spain; 3 Department of Gastroenterology, Complexo Hospitalario Universitario de Ourense, Instituto de Investigación Sanitaria Galicia Sur, CIBEREHD, 32005 Ourense, Spain; 4 Department of Gastroenterology, Hospital Universitario de Burgos, 09006 Burgos, Spain; 5 Department of Gastroenterology, Hospital Donostia/Instituto Biodonostia, Centro de Investigación Biomédica en Red de Enfermedades Hepáticas y Digestivas (CIBEREHD). Universidad del País Vasco UPV/EHU, 20014 San Sebastián, Spain; 6 Department of Gastroenterology, Hospital Clínico Universitario, IIS Aragón, University of Zaragoza, CIBEREHD, 50009 Zaragoza, Spain; 7 Gastroenterology Unit, Hospital General Universitario de Alicante, 03010 Alicante, Spain; 8 Department of Gastroenterology, Complexo Hospitalario Universitario de Vigo, 36214 Vigo, Spain; 9 Department of Gastroenterology, Hospital Universitario de Canarias, Universidad de La Laguna, Instituto Universitario de Tecnologías Biomédicas (ITB) & Centro de Investigación Biomédica de Canarias (CIBICAN), 38320 San Cristobal de La Laguna, Tenerife, Spain; 10 Advanced Marker Discovery (AMADIX), Acera de Recoletos 2, 47004 Valladolid, Spain

**Keywords:** biomarker, tumor marker, plasma, serum, colon cancer, qRT-PCR, reference miRNA, endogenous control, normalization, non-invasive diagnosis

## Abstract

**Background:** Circulating microRNA (miRNA) analysis is a growing research field. However, it usually requires an endogenous control or housekeeping (HK) in order to normalize expression of specific miRNAs throughout different samples. Unfortunately, no adequate HK for circulating miRNA analysis is still known in the colorectal cancer (CRC) context whereas several have been suggested. Hence, our aims were to validate the previously suggested miR-1228-3p as HK for CRC studies, to compare its suitability with the widely used miR-16-5p, and to evaluate the influence of hemolysis on both miRNAs. **Methods:** We analyzed by quantitative reverse-transcription polymerase chain reaction (qRT-PCR) the expression of miR-1228-3p, miR-16-5p and the spike-in cel-miR-39 in a set of 297 plasmas (92 CRC, 101 advanced adenomas -AA-, and 100 controls) and 213 serum samples (59 CRC, 74 AA and 80 controls). We also analyzed both miRNAs depending on the hemolysis degree in 7 plasmas and 31 serums. **Results:** Levels of miR-1228-3p and miR-16-5p did not show significant differences between groups although miR-16-5p exhibited more variability in plasma and serum samples. Importantly, the combination of cel-miR-39 and miR-1228-3p was the most stable one. Moreover, we observed that miR-16-5p was significantly influenced by hemolysis in contrast with miR-1228-3p that exhibited no correlation with this confounding factor in both biofluids. **Conclusion:** MiR-1228-3p has been validated as an adequate endogenous control for circulating miRNA analysis in CRC and AA liquid biopsies.

## 1. Introduction

MicroRNAs (miRNAs) are small endogenous non-coding RNAs that regulate thousands of mRNAs at posttranscriptional level [1]. Over the last few years, miRNA expression studies have increased due to its relevant function in different biological and pathological processes [2,3]. In fact, they play relevant roles in cancer as tumor suppressors or oncogenes [4]. Moreover, they are found circulating in different body fluids such as plasma, serum or urine; making them potential non-invasive biomarkers, as well as the base for new treatment therapies [5,6,7].

Colorectal cancer (CRC) is one of the cancers with highest incidence worldwide that could benefit from non-invasive biomarkers, such as circulating miRNAs, for their early detection due to it follows a stepwise development starting with a benign precursor lesion called advanced adenoma (AA) in which early molecular changes are observed [8,9,10].

Indeed, quantification of miRNA levels can be performed by different techniques [11,12] being quantitative reverse-transcription polymerase chain reaction (qRT-PCR) one of the most commonly used due to its high sensitivity and specificity [13]. The data obtained with qRT-PCR requires a normalization process in order to reduce the potential technical bias, which can be caused by pre-analytical or technical factors arising from miRNA extraction to the amplification process. However, a universally accepted housekeeping (HK) suitable for all kind of samples and conditions does not exist, and there is still no standard normalization method for plasma/serum miRNA analysis in CRC. These facts result in lack of consistency in some findings from similar studies about the same disease [14,15].

In addition, an important interfering factor working with miRNAs in biofluids such as plasma or serum is the presence of hemolysis that can affect expression levels of some miRNAs [16,17] and, therefore, this has to be taken into account when choosing a HK miRNA for these analyses.

Furthermore, a reliable HK miRNA has to be abundant enough in the sample of interest and stable between groups. Thus, each kind of sample and disease should have its appropriate HK miRNA since miRNA expression patterns differ among different samples and diseases [18]. Recently, miR-1228-3p has been suggested as a potential HK for plasma samples in different cancers, including CRC [19,20].

Therefore, the aim of our study was to confirm whether miR-1228-3p could be an adequate endogenous control for circulating miRNA expression analysis in plasma and serum in large cohorts of CRC patients, as well as whether it could be also suitable when analyzing samples coming from patients with pre-neoplastic lesions (AA). We also compared these results with those obtained with miR-16-5p, one of the most commonly used HK in this context despite having various inherent limitations. Finally, we also evaluated the influence of the degree of hemolysis in the expression of both HK candidates.

## 2. Patients and Methods

### 2.1. Patients

Plasma set: plasmas from 297 individuals (96 CRC, 101 AA and 100 controls) from 8 different Spanish hospitals were analyzed. The clinico-pathological status of participants from the plasma set is shown in Table 1.

Serum set: serums from 213 individuals (59 CRC, 74 AA and 80 controls) provided by the Hospital Clinicof Barcelona were also analyzed. Table 2 shows clinicopathological status of participants from the serum set.

None of patients included in this study had received chemo- or radiotherapy before sample collection. The study was approved by the Institutional Ethics Committees of each hospital and written informed consent was obtained from all participants in accordance with the Declaration of Helsinki.

### 2.2. Samples

Peripheral blood from participants was drawn before colonoscopy either in 10 mL BD Vacutainer^®^ K_2_EDTA tubes (Becton Dickinson, NJ, USA) or in 10 mL BD Vacutainer^®^ Plus plastic serum tubes (Becton Dickinson). Plasma was obtained after double centrifugation. Serum was obtained by one centrifugation after incubation for 30 min at room temperature to form the clot. Plasma and serum samples were stored at −80 °C until RNA extraction.

### 2.3. Hemolysis Assessment

Hemolysis degree of plasma and serum samples was measured by spectrophotometer at optical density of 414 nm (OD_414nm_). We considered non-hemolytic samples those with OD_414nm_ below or equal to 0.25, the rest of samples were discarded. For assessment of hemolysis influence on HK miRNA expression, 7 plasma and 31 serum samples were chosen (3 and 14, respectively, were regarded as hemolytic for showing an OD_414nm_ > 0.25).

### 2.4. RNA Extraction

Total RNA, including small RNAs, was isolated from 500 µL of plasma or serum by using mirVana™ PARIS™ kit (Thermo Fisher Scientific, Inc., Waltham, Massachusetts, USA) according to the manufacturer’s protocol. Previous to RNA extraction, 5 µL of cel-miR-39 at 5 nM was added as spike-in exogenous control. Final elution volume was 30 µL. RNA was kept at −80 °C until miRNA expression analysis was done.

### 2.5. miRNA Expression Analysis

miRNA expression was assessed by qRT-PCR using TaqMan^®^ MicroRNA assays (Thermo Fisher Scientific, Inc.). Briefly, RNA was retrotranscribed by using TaqMan^®^ MicroRNA reverse transcription kit (Thermo Fisher Scientific, Inc.). After that, quantitative PCR was performed in a Viia7^®^ Real Time PCR system (Thermo Fisher Scientific, Inc.) according to the manufacturer’s protocol.

Cel-miR-39 expression was used to normalize differences in extraction efficiency. Each point was assessed in triplicate.

### 2.6. Statistical Analysis

Samples with missing **cycle threshold** (Ct) value in at least one of the analyzed miRNAs were removed. Interquartile range (IQR) and standard deviation (SD) were calculated for each HK miRNA to evaluate the variability. Samples that had a Ct value of Ct ± (IQR × 1.5) were considered as outliers and were discarded for the expression analysis. Student’s *t*-test was performed to evaluate miRNA expression differences between groups (CRC vs Control; AA vs Control; CRC vs AA). Regarding influence of hemolysis, we compared miRNA expression between hemolytic and non-hemolytic samples. Existence of correlation between miRNA expression (miR-1228-3p or miR-16-5p) and hemolysis was assessed by Pearson correlation analysis. *p*-value ≤ 0.05 was regarded as significant. All statistical analysis and graphics were performed by IBM^®^ SPSS Statistics^®^ 23 software (New York, NY, USA).

## 3. Results

### 3.1. Expression Levels of Housekeeping (HK) miRNA Candidates in Plasma Samples

**Figure 1:**
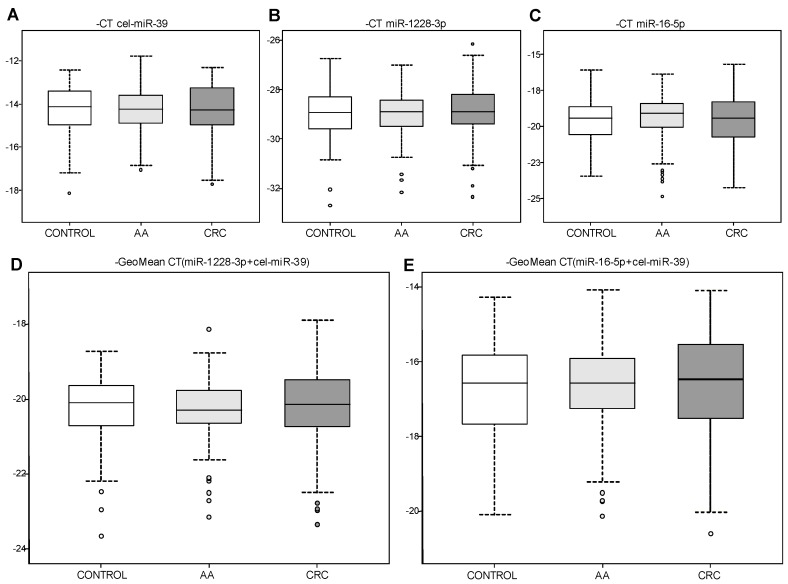
Expression levels of candidate endogenous control miRNAs in plasma samples from a CRC cohort by quantitative reverse-transcription polymerase chain reaction (qRT-PCR). Minus Ct of spike-in (**A**) cel-miR-39, candidate endogenous controls (**B**) miR-1228-3p and (**C**) miR-16-5p are depicted as box-plots. Minus geometrical mean Ct of each endogenous control with cel-miR-39 are also shown (**D** and **E**). There is no significant difference in the expression levels of cel-miR-39, miR-1228-3p and miR-16-5p between control individuals (control; white box; *n* = 98), individuals with advanced adenomas (AA; light grey box; *n* = 99) and colorectal cancer patients (CRC; dark grey box; *n* = 90).

Ten plasma samples were excluded from the subsequent analysis because they showed no Ct value in at least one of the analyzed miRNAs. Thus, 287 plasma samples (90 CRC, 99 AA and 98 controls) were finally analyzed. Cel-miR-39 expression results exhibited a mean Ct of 14.24 and standard deviation (SD) of 1.11 (Figure 1A). Both HK miRNA candidates assessed, miR-1228-3p and miR-16-5p, showed reliable Ct levels. More specifically, miR-1228-3p had a mean Ct of 28.93 with SD = 1.01 (Figure 1B) and miR-16-5p showed a mean Ct of 19.49 with SD = 1.62 (Figure 1C). MiR-1228-3p appeared among a 6.54 Ct range [26.16–32.70] and miR-16-5p among a 9.18 Ct range [15.68–24.85]. Accordingly, miR-1228-3p exhibited an IQR of 1.16 whereas miR-16-5p exhibited an IQR of 2.02, suggesting that the first was less variable between samples. Finally, statistical analysis did not show significant differences between analyzed groups (CRC, AA and controls) in any miRNA although miR-16-5p presented more variability than miR-1228-3p.

A subsequent analysis was performed using cel-miR-39 in combination with each HK miRNA candidate to evaluate if it could improve the normalizing method. The geometric mean (GeoMean) of cel-miR-39 and miR-1228-3p was 20.28 (SD = 0.91) and the IQR was 1.05 indicating this combination was very stable between samples. For cel-miR-39 and miR-16-5p GeoMean was 16.65 (SD = 1.19) and IQR of 1.54 indicating that this combination was more stable than using miR-16-5p alone but less stable than combining miR-1228-3p and cel-miR-39. Statistical analysis did not show significant differences in any of the combinations between groups (Figure 1D,E). Table 3 summarizes the parameters obtained for each normalization method.

### 3.2. Assessment of miR-1228-3p and cel-miR-39 Combination as Normalization Method in Serum Samples

**Figure 2:**
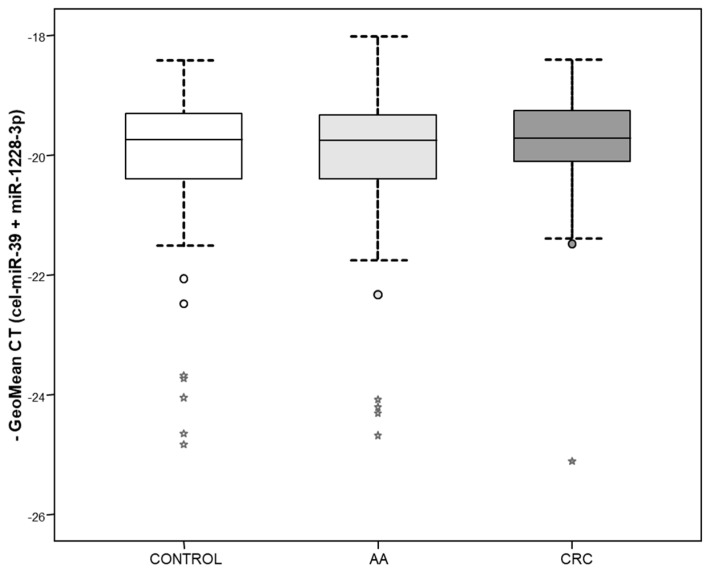
Combination of cel-miR-39 and miR-1228-3p qRT-PCR values in serum samples from a second CRC cohort. Minus geometrical mean Ct is represented as box-plot. There is no significant difference in these values levels between control individuals (control; white box; *n* = 80), individuals with advanced adenomas (AA; light grey box; *n* = 74) and colorectal cancer patients (CRC; dark grey box; *n* = 59).

In order to confirm that the proposed normalization method could also be used in serum samples, we analyzed expression of miR-1228-3p and the spiked-in cel-miR-39 in a large serum set (*n* = 213). Expression levels of cel-miR-39 showed similar pattern than plasma samples being mean Ct of 14.41 (SD = 1.08). MiR-1228-3p also showed similar Ct values than plasma with a mean Ct of 27.70 (SD = 1.65 and IQR = 1.44). As expected, no significant differences were observed between groups for these 2 miRNAs. The combination of both showed GeoMean = 19.97, SD = 1.23 and IQR = 1.03. Accordingly, it did not show significant differences between groups (Figure 2). Thus, results in serum were similar to those obtained in plasma indicating that the combination of miR-1228-3p and cel-miR-39 could also be a good strategy to normalize miRNA expression in serum samples.

### 3.3. Assessment of Hemolysis Interference on HK miRNA Expression

**Figure 3:**
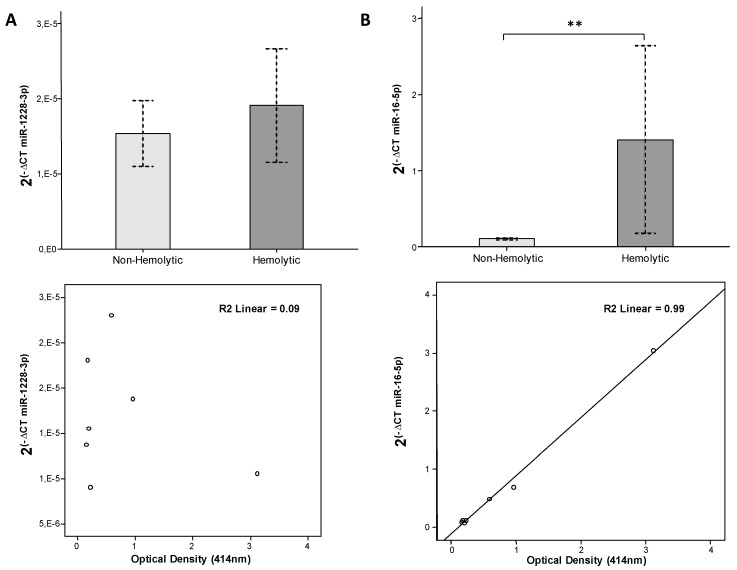
Influence of hemolysis in miR-1228-3p and miR-16-5p plasma levels. qRT-PCR data are represented as 2^(−CT)^ normalized by cel-miR-39 for (**A**) miR-1228-3p and (**B**) miR-16-5p. Correlation between expression of these miRNAs and optical density (OD) measured at 414 nm are also shown. Hemolytic plasma samples (*n* = 3) regarded as OD_414nm_ > 0.25; non-hemolytic plasma samples (*n* = 4) regarded as OD_414nm_ ≤ 0.25. *p*-value ≤ 0.01 is depicted as **.

In order to know if miR-16-5p and miR-1228-3p expression was affected by hemolysis and which of them had more variability due to this factor we compared their levels in plasma (*n* = 7) and serum (*n* = 31) samples with different degrees of hemolysis. Results showed that miR-1228-3p levels in plasma were not significantly affected by hemolysis (Figure 3A). On the contrary, miR-16-5p levels exhibited high correlation with hemolysis (R^2^ = 0.99; *p*-value < 0.001) and were significantly higher in hemolytic plasma samples than in non-hemolytic ones (Figure 3B).

**Figure 4:**
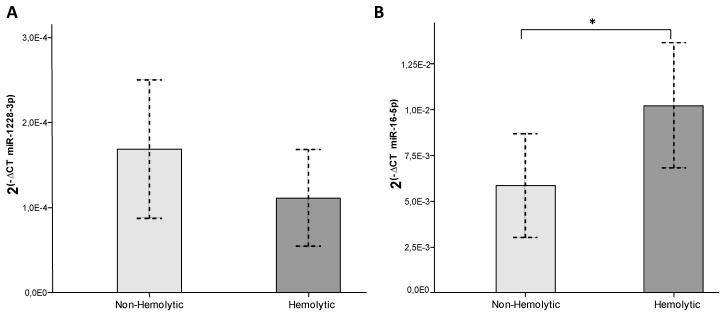
Influence of hemolysis in (**A**) miR-1228-3p and (**B**) miR-16-5p serum levels. qRT-PCR data are represented as 2^(−CT)^ normalized by cel-miR-39. Hemolytic serum samples (*n* = 14) regarded as OD_414nm_ > 0.25; non-hemolytic serum samples (*n* = 17) regarded as OD_414nm_ ≤ 0.25. *p*-value ≤ 0.05 is depicted as *.

Similarly, the same tendency was observed in serum samples. MiR-1228-3p showed no significant differences between hemolytic and non-hemolytic serum samples while miR-16-5p increased significantly in the hemolytic ones (Figure 4). Thus, miR-16-5p and not miR-1228-3p expression levels can be influenced by hemolysis present in samples and can introduce bias if used to normalize other miRNAs expression. Therefore, a part from being the most stable normalizing method, the combination of cel-miR-39 and miR-1228-3p is not influenced by hemolysis.

## 4. Discussion

Over the last few years, miRNA studies have increased and pointed out these molecules as excellent biomarkers for different pathologies [21]. Importantly, miRNAs have especially been found altered in different cancers [22,23]. These molecules could have an important role helping clinicians to detect cancer at early stages as well as guide treatment response and surveillance [24]. Furthermore, miRNAs were also found in circulating body fluids making them potential non-invasive biomarker candidates [25]. However, there are several pre-analytical and analytical factors that can interfere with circulating miRNA measurements [26,27]. As a consequence, standardization of the analytical method is still lacking, thus producing discrepancies in the results obtained by different methods. This fact represents a drawback to develop new non-invasive biomarkers based on miRNAs and makes obtaining reliable results difficult. Quantitative reverse-transcription PCR (qRT-PCR) is the most commonly used technique to quantify miRNA expression [28]. However, Ct values have to be normalized with one or several reference genes or HKs to obtain reliable results; thus enabling to reduce the bias due to differences between samples in miRNA extraction efficiency, reverse-transcription and amplification. In fact, the use of inadequate HK can generate misleading results [29,30]. Unfortunately, there are still not enough studies validating potential HK miRNAs to be used in qRT-PCR analysis of circulating miRNAs in CRC.

The main aim of our study was to validate a previously suggested HK miRNA candidate for circulating miRNA studies in CRC patients, in a large set of plasma samples, and compare it with one of the most commonly used HK miRNAs in this setting such as miR-16-5p. Secondly, we wanted to also confirm the suitability of miR-1228-3p as HK miRNA in serum samples. We found in the literature that Hu J et al. described that miR-1228-3p seemed to be stable in plasma samples from different kind of tumors, including CRC [19]. After that, Danese, E. et al. confirmed the stability of this miRNA in plasma, exosomes and tissue from 20 CRC patients [20]. However, validation in a large cohort of patients also including patients with pre-neoplastic lesions such as advanced adenomas (AA), provided confirmation of its suitability in serum samples and assessment of the influence of one of the most confounding factors in this setting (i.e., hemolysis), were still lacking.

Therefore, we analyzed circulating levels of miR-1228-3p and miR-16-5p in two large cohorts of CRC patients (also containing patients with AA), one consisting of plasma and the other of serum samples. Then, we compared their levels in hemolytic samples with non-hemolytic ones to assess the influence of this common phenomenon on the aforementioned HK candidates. Our results showed that both miR-16-5p and miR-1228-3p were found with consistent expression in all analyzed plasma samples and no significant differences were detected between CRC, AA or control patients. However, miR-1228-3p showed less variability between samples than miR-16-5p as indicated by the corresponding IQR and SD. Moreover, taking into account that normalizing only with an endogenous control any possible bias due to differences in miRNA extraction and technical efficiency is not considered [31], we also added a non-human exogenous control, cel-miR-39, before RNA extraction. We then considered to combine both exogenous and endogenous controls and calculated the geometric mean of cel-miR-39 and each one of the endogenous controls assessed. Both combinations (cel-miR-39 + miR-16-5p or cel-miR-39 + miR-1228-3p) were more stable than using the respective endogenous control alone, but the combination with miR-1228-3p was the most stable.

In addition, we validated the stability of the proposed HK combination cel-miR-39 + miR-1228-3p in a large serum set of CRC and AA patients, and healthy individuals. As expected, it also showed very low variability among all serum samples and no differences between groups.

Furthermore, bearing in mind that presence of hemolysis can affect some plasma and serum miRNA levels due to miRNA contamination coming from blood cells [16,17], it is extremely important that our HK miRNA that we will use to normalize results is not influenced by this factor, otherwise some bias could be introduced resulting in inaccurate results and conclusions. Indeed, there are some studies that found miR-16-5p among the top 10 miRNAs expressed in erythrocytes [32,33]. Consistently, we observed that hemolysis had a significant influence on miR-16-5p levels in plasma and serum samples, thus adding another drawback to the use of this miRNA as HK in this setting. Conversely, miR-1228-3p did not show any relation with the hemolysis degree and no significant difference was found between hemolytic and non-hemolytic samples.

Finally, an ideal HK miRNA to be used in CRC studies should not be involved in cancer biology. However, for the widely used HK miRNA miR-16-5p there are lots of reports demonstrating its deregulation and implication in cancer processes, including CRC, regulating onco/tumor suppressor genes and having an important role in cell growth in cancer [34,35,36]. By contrast, no relation with colon cancer processes has been found for miR-1228-3p that has been only related with the regulation of some metabolic pathways and organ morphology [19].

In conclusion, we suggest that the combination of an exogenous control (for instance, cel-miR-39) and the endogenous control miR-1228-3p could be a good strategy to normalize miRNA expression analysis obtained by qRT-PCR in plasma and serum from CRC or AA patients.

## Figures and Tables

**Table 1:** Clinicopathological characteristics of patients included in the plasma set. Tumor stage was classified according to staging system of American Joint Committee on Cancer (AJCC), and location according to proximal or distal classification. Adenomas characteristics are explained as dysplasia grade, villous component and size of the largest one. (SD: standard deviation; CRC: colorectal cancer; AA: advanced adenoma; control; healthy volunteers).

	Total	CRC	AA	Control
Number	297	96	101	100
Mean Age (SD)	65 (11.5)	73 (10.6)	63 (9.4)	60 (11.1)
**Gender**				
Male	174	50	73	51
Female	123	46	28	49
**CRC Features**				
	**TNM Stage**			
I		20		
II		23		
III		34		
IV		14		
Unkown		5		
	**Location**			
Proximal		37		
Distal		59		
**AA Features**				
Mean Size (mm) (SD)			20 (11.8)	
N AA Mean (SD)			3 (3.4)	
N AA ≥ 10 mm			93	
High-grade dysplasia			38	
Villous Component			41	
Unkown			5	

**Table 2:** Clinicopathological characteristics of patients included in the serum set. Tumor stage was classified according to staging system of American Joint Committee on Cancer (AJCC), and location according to proximal or distal classification. Adenomas characteristics are explained as dysplasia grade, villous component and size of the largest one. (SD: standard deviation; CRC: colorectal cancer; AA: advanced adenoma; control: healthy volunteers).

	Total	CRC	AA	Control
Number	213	59	74	80
Mean Age(SD)	62 (5,5)	62 (5,4)	62 (5,6)	62 (5,6)
**Gender**				
Male	150	44	51	55
Female	63	15	23	25
**CRC Features**				
	**TNM Stage**			
I		30		
II		13		
III		14		
IV		2		
	**Location**			
Proximal		18		
Distal		41		
**AA Features**				
Mean size (mm) (SD)			14 (7,2)	
N AA Mean (SD)			3 (1,8)	
N AA ≥ 10 mm			63	
High-grade dysplasia			26	
Villous component			27	

**Table 3:** Summary results of different housekeeping (HK) expression in plasma and serum samples from two CRC cohorts by qRT-PCR. (IQR, interquartile range; SD, standard deviation).

Plasma (*n* = 287)	Mean Ct	Range Ct (Ct_min_ − Ct_max_)	IQR	SD
miR-1228-3p	28.9	6.5 (26.2–32.7)	1.16	1.0
miR-16-5p	19.5	9.2 (15.7–24.9)	2.02	1.6
Cel-miR-39 + miR-1228-3p	20.3	5.8 (17.9–23.7)	1.05	0.9
Cel-miR-39 + miR-16-5p	16.7	6.5 (14.1–20.6)	1.54	1.2
**Serum (*n* = 213)**				
miR-1228-3p	27.7	11.3 (24.7–36.1)	1.44	1.65
Cel-miR-39 + miR-1228-3p	20.0	7.1 (18.0–25.1)	1.03	1.23

## Abbreviations

AA	advanced adenomas
CRC	colorectal cancer
miRNA	microRNA
qRT-PCR	real-time quantitative reverse transcription PCR
HK	housekeeping
OD	optical density
IQR	interquartile range
SD	standard deviation
CT	cycle threshold
Geomean	geometric mean
